# Targeted therapy monitoring of BRAF-V600-mutant Erdheim-Chester disease by fast quantitative whole-body bone CZT-tomoscintigraphies

**DOI:** 10.1186/s41824-022-00160-3

**Published:** 2023-01-13

**Authors:** Saifeddine Melki, Thomas Moulinet, Antoine Verger, Pierre-Yves Marie, Laetitia Imbert, Achraf Bahloul

**Affiliations:** 1 Department of Nuclear Medicine and Nancyclotep, CHRU Nancy, Hôpital de Brabois, Allée du Morvan, 54000 Vandoeuvre-lès-Nancy, Nancy, France; 2 Department of Internal Medicine, CHRU Nancy, 54000 Nancy, France; 3 CNRS UMR 7365, IMoPA, Université de Lorraine, 54000 Nancy, France; 4 IADI, INSERM, UMR 1254, Université de Lorraine, 54000 Nancy, France

**Keywords:** Erdheim-Chester disease, Whole-body bone tomoscintigraphy, CZT-camera, Targeted therapy, [F-18]-FDG PET

## Abstract

Erdheim-Chester disease (ECD) is a rare histiocytosis due to proto-oncogene mutations, primarily affecting the long bones and possibly being treated by novel targeted therapies. ^18^F-FDG PET is a reference technique for ECD assessment. However, we present a case where easier and more objective monitoring of the ECD-related bone metabolism abnormalities under treatment was obtained with the standardized uptake value-based information provided by fast whole-body [Tc-99 m]-HDP bone tomoscintigraphies (QWBT) recorded with a high-sensitivity CZT-camera/computed tomography (CT) hybrid system.

**Supplementary Information:**

The online version contains supplementary material available at 10.1186/s41824-022-00160-3.

## Introduction

Erdheim-Chester disease (ECD) is a histiocytosis due to proto-oncogene mutations, increasingly treated by targeted therapies (Goyal et al. 2020), and primarily affecting the long bones and, more sporadically, cardiovascular and nervous systems and other organs (Mazor et al. 2013). ^18^F-FDG PET is now considered to be the reference technique for whole-body assessment of ECD patients (Kirchner et al. 2021). At the present time, however, fast whole-body recordings and reliable standardized uptake values (SUV) measurements may be obtained not only with PET cameras but also with novel hybrid systems combining: (i) single-photon emission tomography (SPECT) recording through cadmium–zinc–telluride (CZT) detectors with an original 360° ring configuration geometry that is likely to maximize both count sensitivity and image quality and (ii) multi-detector computed tomography (CT) scanners providing the attenuation maps required for SUV quantification (Melki et al. 2020; Bahloul et al. 2021; Mairal et al. 2022; Chevalier et al. 2020). One such system, the high-speed VERITON CZT-SPECT/CT system (Spectrum Dynamics Medical, Caesarea, Israel) was previously shown to provide both reliable SUV measurements and high-quality bone scintigraphy images (Melki et al. 2020; Bahloul et al. 2021; Mairal et al. 2022). A threshold of 7.5 SUV was additionally shown to grossly separate abnormal from normal bone metabolism areas (Bahloul et al. 2021). Bone scintigraphy provided by this VERITON CZT-SPECT/CT system was applied here for the longitudinal monitoring of an ECD patient under treatment.

## Case presentation

**Fig. 1:**
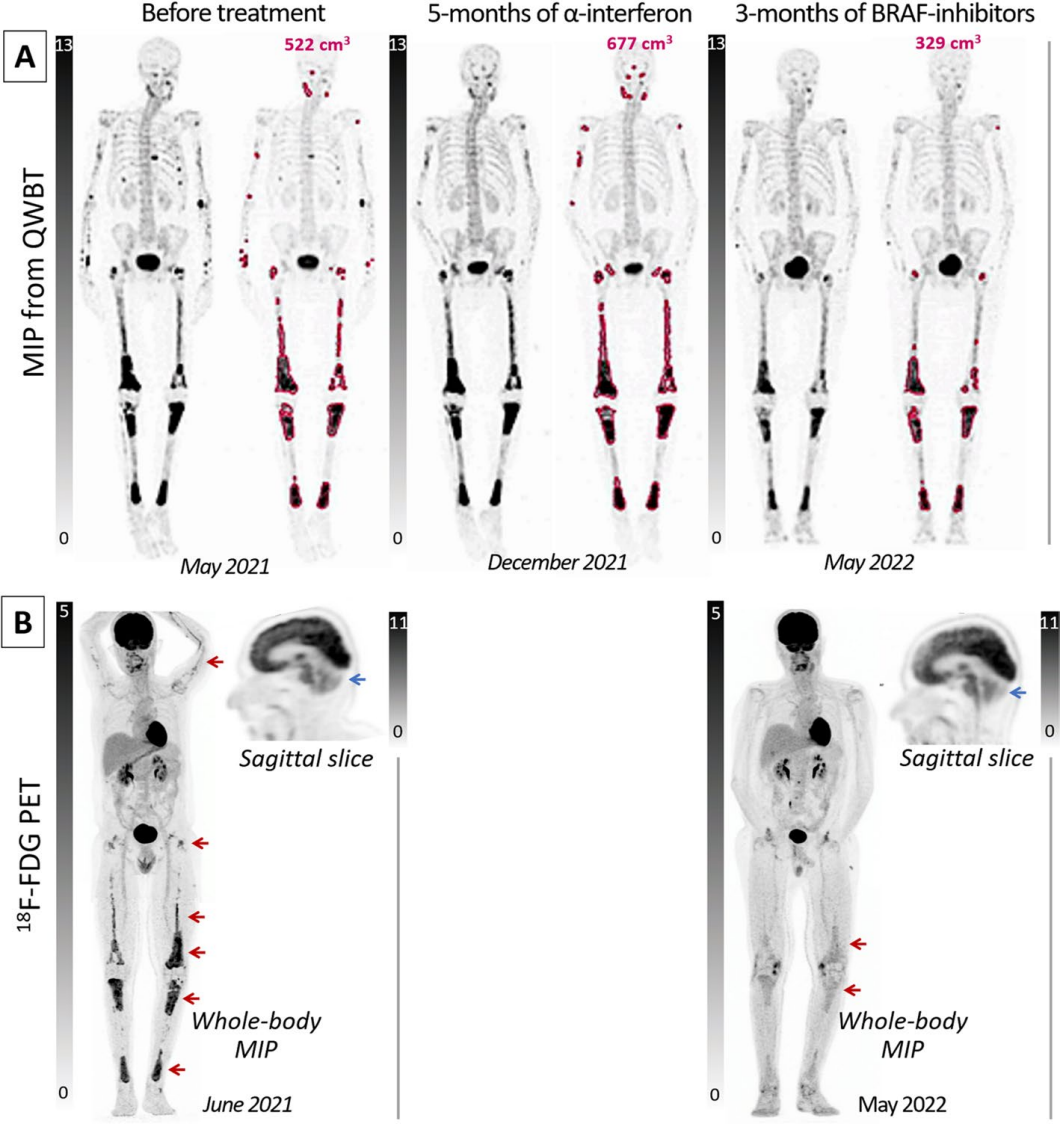
Maximal intensity projection images of QWBT and FDG PET displayed with adapted SUV scaling (respectively 0 to 13 (Bahloul et al. 2021) and 0 to 5), (**A**) before and (**B**) after 5 months of α-interferon treatment followed by 3 months of BRAF inhibitors therapy

This 50-year-old man with cerebellar syndrome was admitted for a pulmonary embolism, and CT scan showed osteocondensation lesions. QWBT was obtained 3 h after ^99m^Tc-hydroxymethylene diphosphonate (HDP) injection, as previously described (Bahloul et al. 2021), and initial QWBT images exhibited rib and sacrum fractures, as well as highly contrasted areas typical of ECD and affecting the mandible and long bones symmetrically, while sparing epiphyseal regions (Mazor et al. 2013). The corresponding maximal intensity projection (MIP) images are displayed in Fig. 1A (May 2021), as well as through rotating cine-loops in Additional file 1, with a conventional 0–13 SUV scaling (Bahloul et al. 2021) and an additional display where red lines represent the limits of hypermetabolism areas [i.e., those > 7.5 SUV (Bahloul et al. 2021)] located on limbs and skull and thus likely due to ECD (Mazor et al. 2013).

Biopsy identified ECD with BRAF-V600 mutation and ^18^F-FDG PET identified a cerebellum hypometabolism (Fig. 1B, June 2021) in line with ECD-related atrophy at MRI (Na et al. 2008). It is of note, however, that the bone abnormalities exhibited a higher contrast on the QWBT than on PET pre-therapeutic images.

On the following QWBTs (Fig. 1A), the volume of ECD-related abnormalities exhibited a slight increase from 522 to 677 cm^3^ in December 2021, despite 5 months of α-interferon treatment, but a subsequent 50% reduction down to 329 cm^3^ on May 2022, after 3 months of more effectively targeted therapy by BRAF inhibitors (Goyal et al. 2020). It is of note that the bone metabolism abnormalities still appeared much easier to delineate on the post-therapeutic QWBT images than on the PET images. This may be explained by the higher bone specificity of ^99m^Tc-HDP than of ^18^F-FDG and to the difference in physiopathological mechanisms imaged by the two radiotracers [i.e., reactive osteosynthesis for ^99m^Tc-HDP vs. inflammatory cells infiltrate for ^18^F-FDG (Ohara et al., 2018)].

## Conclusion

This CZT-SPECT/CT system mimics what is currently obtained with whole-body PET, i.e., the QBWT images recorded with fast 3D acquisitions and displayed with a reliable SUV scale. However, distinctively different to what may be achieved with ^18^F-FDG PET, ^99m^Tc-HDP QBWT enables the use of a simple SUV-based threshold methodology to grossly delineate abnormal areas due to the high specificity of the bone scintigraphy tracer. These properties result in easy and objective monitoring of the changes in bone abnormalities under treatment in the ECD patient presented herein. It is likely that these properties could also be helpful for the monitoring of many other diseases affecting the skeleton.

## Supplementary Information

**Additional file 1**. Maximal Intensity Projection (MIP) images of QWBT displayed with adapted SUV scaling (0 to 13) through rotating cine-loops before and after 5 months of α-interferon treatment followed by 3 months of BRAF inhibitors therapy.

## Data Availability

Not applicable.
